# Population structure and zoonotic potential of *Cryptosporidium parvum* in Italy inferred using a multi-locus sequence typing scheme

**DOI:** 10.1186/s13071-025-07236-6

**Published:** 2026-01-24

**Authors:** Marianna Ascierto, Antonio Di Grazia, Francesco Celani, Nicoletta D’Avino, Luciana Petrullo, Maria Grazia Coppola, Simone M. Cacciò

**Affiliations:** 1 UOC Microbiology and Virology, Azienda dei Colli of Naples, “D. Cotugno” Hospital, Naples, Italy; 2 Department of Experimental Medicine, University of Campania “Luigi Vanvitelli”, Naples, Italy; 3 Department of Infectious Diseases, Istituto Superiore di Sanità, Rome, Italy; 4 Istituto Zooprofilattico Sperimentale delle Marche e dell’umbria, Perugia, Italy

**Keywords:** *Cryptosporidium parvum*, Italy, Multi-locus genotyping, Population structure, Zoonosis

## Abstract

**Background:**

The genetic variability of a large collection of European samples of the zoonotic pathogen *Cryptosporidium parvum* has been recently explored on the basis of a novel multi-locus sequence typing (MLST) scheme. In this work, we assessed the usefulness of this scheme to type *C. parvum* samples from Italy, a country where this pathogen is widespread and associated with human infections.

**Methods:**

Polymerase chain reaction (PCR) and sequencing for the eight markers of the MLST scheme were performed on 31 human- and 21 animal-derived *C. parvum* samples. MLST data from 27 samples of animal origin previously sequenced at the genome level were also included. Sequence data for the glycoprotein 60 (*gp60*) gene were also generated. Phylogenetic and cluster analyses were conducted.

**Results:**

Full genotyping data were obtained for 72 of 79 samples, and 39 different profiles were categorized, 28 of which were found in individual samples (singletons). A new allele was found at the marker on chromosome 2 in a human-derived sample. When compared with the 154 profiles previously described in Europe, 30 of the 39 profiles (76%) were found to be restricted to Italy, a result compatible with a model of isolation by distance, with geographically structured populations. Analysis of the *gp60* sequences identified 19 different subtypes among the 55 samples belonging to family IIa, and 7 different subtypes among the 16 samples belonging to family IId. Phylogenetic and haplotype analyses did not identify clusters related to the host, the geographic origin (i.e., the Italian regions), or the time of collection of the samples but did identify two different populations, mirroring data obtained from whole genome comparative analyses.

**Conclusions:**

The MLST scheme appears to be a promising method for genotyping *C. parvum* samples, as it provided higher discrimination compared with *gp60* and enabled the recognition of the two major populations circulating in Europe and in Italy.

**Graphical Abstract:**

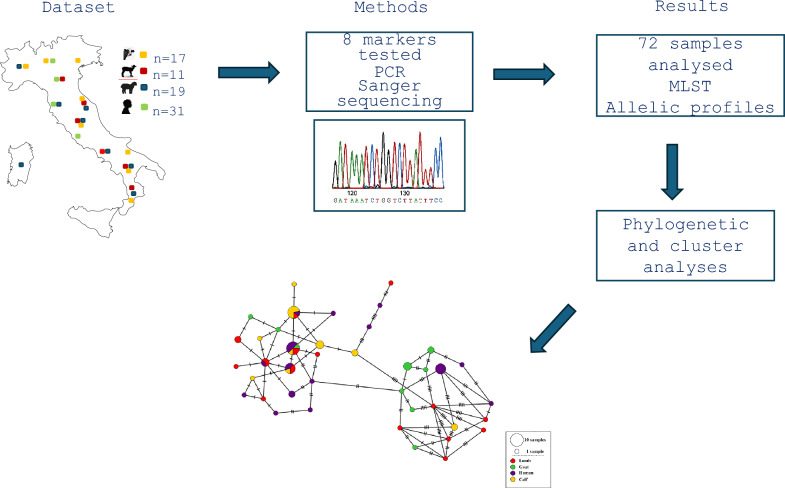

**Supplementary Information:**

The online version contains supplementary material available at 10.1186/s13071-025-07236-6.

## Background

The ubiquitous Apicomplexan parasite *Cryptosporidium parvum* causes a diarrheal disease, known as cryptosporidiosis, in both humans and young ruminants [1]. Infection starts with the ingestion of oocysts, which are shed with the feces of infected hosts and can persist in the environment and maintain their infectivity for long periods. The epidemiology of the infection is complex, as both direct (human-to-human, animal-to-human) and indirect (through ingestion of contaminated water or food) transmission routes exist [2].

Cryptosporidiosis is a notifiable disease under surveillance at the European level. In the latest report from the European Centre for Disease Prevention and Control (ECDC), 4164 cases were officially reported by 24 European countries through the European Surveillance System (TESSy), with higher prevalence rates among children aged 0–4 years [3].

In Italy, however, human cryptosporidiosis is not yet included in the list of notifiable diseases, and therefore, cases are likely to be strongly underdiagnosed and underreported. The limited information available, mostly derived from historical surveys in immunocompromised individuals or case reports, indicates that human infections are caused by *C. parvum* [4, 5]. More information is available on the animal reservoir, mainly young ruminants, where the circulation and clinical impact of *C. parvum* is well established [6, 7]. The few environmental studies conducted have shown that water matrices, especially wastewater, are frequently contaminated by the parasite, and a large waterborne outbreak was described in Northern Italy, with more than 80 cases identified [8].

Molecular methods are needed to understand transmission pathways and distinguish epidemic clusters (e.g., outbreaks) from sporadic cases [9]. Typing of *Cryptosporidium* is based either on sequencing of one or more gene markers or on fragment typing of loci containing variable number of tandem repeats (VNTR). Recently, a scheme based on 7 VNTR loci has been shown to be a robust, informative, and economical to type *C. parvum* samples [10]. We recently developed a new multi-locus sequence typing (MLST) scheme for *C. parvum* [11] and discussed its application on a global European scale.

In this study, we aimed at assessing the discriminatory power of this scheme by focusing on a collection of *C. parvum* samples from a single country, Italy, where this parasite is widespread and responsible for most human cases.

## Methods

### Parasite isolates

Samples of *C. parvum* of human origin (*n* = 11) were collected at the Cotugno Hospital (Naples, Italy) during 2022–2025 and were anonymized before being sent to the Istituto Superiore di Sanità (ISS) in Rome for molecular analysis. Five human-derived samples collected during a waterborne outbreak of cryptosporidiosis that occurred in 2019 in Italy [8] were included. Additional samples from human sporadic cases (*n* = 15), collected during 2000–2011 and maintained as frozen stocks at ISS, were also included.

Samples of *C. parvum* derived from young ruminants (calf, lamb, and goat kid) were collected by the Istituto Zooprofilattico Sperimentale dell’Umbria e delle Marche (Perugia, Italy) during 2011–2022 (one sample was collected in 1990). The list of samples used for this study along with the available metadata is provided in Electronic Supplementary Material (ESM) Table S1 in Additional file 1.

### DNA extraction, PCR, and sequencing

Fresh fecal samples of human and animal origin (about 2 g) were mixed with water, filtered through a mesh, and centrifuged for 10 min at 2700 rpm. The washing step was repeated three times and the fecal sediment suspended in approximately 5 mL of water. One sample consisted of a microscopy slide prepared from the feces of an infected lamb and collected during an investigation of a human case of cryptosporidiosis [5]. In this case, oocysts were recovered from the slide by scraping the surface and washing it with tris(hydroxymethyl)aminomethane–ethylenediaminetetraacetic acid (TE) buffer (tris(hydroxymethyl)aminomethane [Tris] 10 mM, ethylenediaminetetraacetic acid [EDTA] 1 mM; pH = 8.0).

Prior to DNA extraction, an aliquot (about 200 µL) of fecal sediment was subjected to five cycles of freezing in liquid nitrogen and thawing at 56 °C. The same procedure was applied to the suspension of oocysts recovered from the slide. DNA extraction was performed using the QIAmpStool DNA kit (Qiagen) following the manufacturer’s instructions.

Experimental conditions for PCR amplification and sequencing of the eight markers were as previously described [11]. Briefly, nested PCR reactions were performed in a final volume of 30 µL consisting of 15 µL of 2X HotStartTaq Master mix (Qiagen), 1 µL of each primer (at 10 pmol/µL), 3 µL of DNA, and 10 µL of sterile water. Amplification of a ~800 bp fragment of the glycoprotein 60 (*gp60*) gene was performed according to a published protocol [12].

Amplification products were visualized on a capillary gel electrophoresis apparatus (QIAxcel, Qiagen) and purified using spin columns (QIAquick PCR purification kit, Qiagen). Bidirectional Sanger sequencing of PCR amplicons was performed by a commercial service (Eurofins Genomics), and the resulting trace files were analyzed using the DNASTAR Lasergene v. 17 software.

### Phylogenetic and cluster analyses

The sequences of the eight markers were concatenated manually, and a multiple sequence alignment was generated using ClustalX 2.0 [13]. The phylogenetic analysis was conducted using the software MEGA11 [14]. A neighbor-joining tree was inferred using the Tamura three-parameter model, while the robustness of the tree topology was evaluated using 1000 bootstrap replicates.

Each distinct sequence (i.e., each allele) at each marker was allocated an integer number, and this was repeated for all eight markers. Next, the allele numbers at each marker were concatenated to define the multi-locus sequence type (MLST) that characterized each sample. The resulting allelic matrix was imported, along with available metadata, into the PopArt software [15], and minimum spanning trees were generated (Table 1).

**Table 1:** Host and geographical origin of the *C. parvum* samples included in this study

Host	Northern Italy	Central Italy	Southern Italy
Human^a^	9	7	11
Calf	6	3	8
Lamb	2	9	8
Goat kid	1	6	5
Total	18	25	32

^a^The geographic origin was unknown for four human samples

## Results

### Application of the MLST scheme

PCR amplification and sequencing of the eight markers in the MLST scheme allowed us to collect full genotyping data for 45 of the 52 *C. parvum* samples tested, precisely 5 from calves, 7 from lambs, 9 from goat kids, and 24 from humans (ESM Table S2 in Additional file 2). The seven samples for which complete genotyping data could not be generated were of human origin and have been frozen for many years, possibly affecting their performance in PCR experiments (Additional file 2: Table S2). One sample from a goat kid (IT-G326) showed a mixed sequencing profile at the marker on chromosome 8, compatible with the presence of two alleles (1 and 6).

We next added the genotyping data from 27 samples of animal origin, previously analyzed by whole genome sequencing [16], and assembled a dataset of 72 fully genotyped samples. In this dataset, the number of alleles varied between three (for markers on chromosome 1 and 3) and seven (for the marker on chromosome 4). Compared with the alleles previously identified in Europe, a new allele (allele 10) was identified at the chromosome 2 marker in the human-derived sample H34 (ESM Table S2 in Additional file 2). The combination of alleles at the eight makers defined 39 different profiles, 28 of which were found in individual samples (singletons). A few profiles were found in multiple samples from multiple hosts and from different Italian regions (ESM Table S2Additional file 2). As expected, five randomly selected samples from a human waterborne outbreak that occurred in Italy showed the same profile, which was not found in any other human or animal sample tested (ESM Table S2 in Additional file 2).

Overall, of the 39 profiles found among Italian samples, 30 (76%) were not previously identified in samples from other European countries and were therefore restricted to Italy.

### Phylogenetic and cluster analyses

A multiple alignment of the eight concatenated marker sequences, totaling 4155 base pairs and comprising 38 informative single-nucleotide polymorphism (SNPs), was used to infer phylogenetic relationships. A neighbor joining tree (ESM Fig. S1 in Additional file 3) did not reveal strongly supported clusters of samples from a single host or from a specific geographic origin (i.e., an Italian region).

**Fig. 1:**
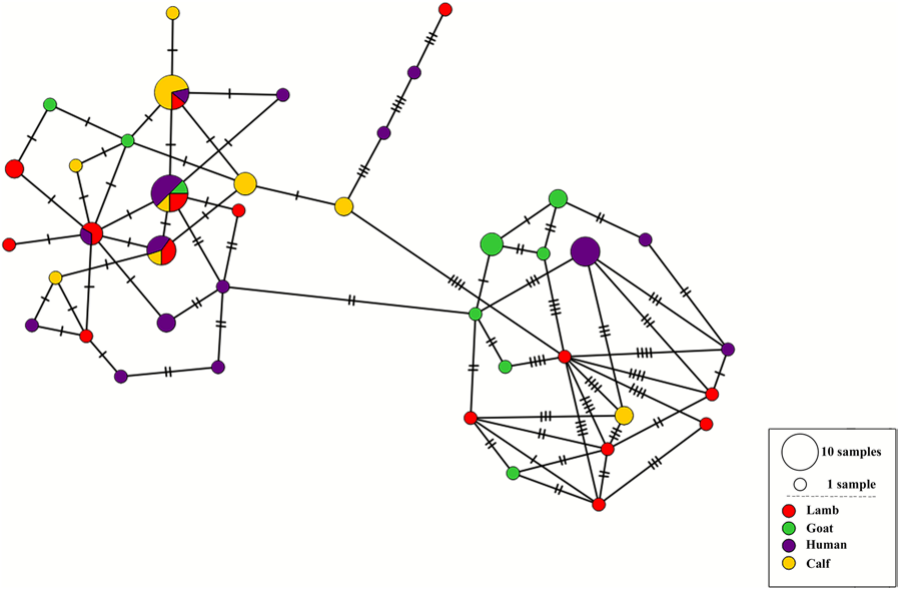
Haplotype network showing the distribution of the 39 MLSTs found in 72 *C. parvum* samples from Italy. Samples are labelled by host species (human, cattle, sheep, goat)

We next used an allelic matrix to generate a minimum spanning tree network and again observed no complete clustering by host (Fig. 1), geographical origin (ESM Fig. S2 in Additional file 4), or year of collection (ESM Fig. S3 in Additional file 5) of the samples. For these analyses, the sample IT-G3266 was analyzed as composed of two populations, with allele 1 or allele 6 at the marker on chromosome 8.

### Comparison with *gp60* sequence data

**Fig. 2:**
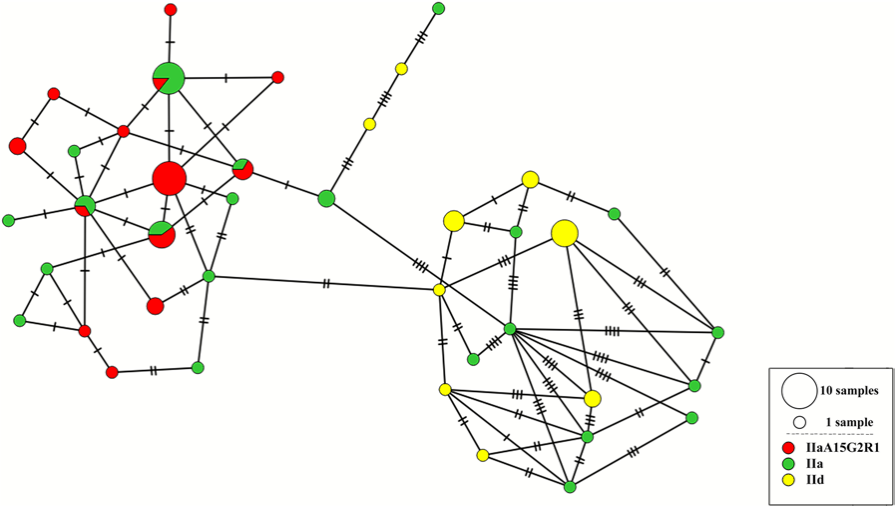
Haplotype network showing the distribution of the IIa and IId *gp60* families over the MLST profiles found in 72 Italian samples. Note the clustering of the 25 samples having the *gp60* subtype IIaA15G2R1 (red)

We determined the sequence of a *gp60* gene fragment (about 800 bp) for the 72 fully genotyped samples (including sample IT-G326, categorized as a potential mixed infection by the MLST scheme). The sequence analysis revealed that 55 samples belonged to the IIa family while the remaining 16 belonged to the IId family (ESM Table S2 in Additional file 2). There were 19 different subtypes in the family IIa, among which subtype IIaA15G2R1 was the most common, being found in 25 samples and in the four host species (ESM Table S2 in Additional file 2). Among the seven different subtypes in family IId, subtype IIdA17G1 was found in five samples from goat kids, whereas subtype IIdA25G1 characterized the five human samples from a waterborne outbreak (ESM Table S2 in Additional file 2). A minimum spanning tree network based on *gp60* families showed two main groups, one comprising only samples of the IIa family, including all samples with the common IIaA15G2R1 subtype, while the second comprised all samples of the IId family along with nine samples of the IIa family (Fig. 2).

Samples sharing the same *gp60* subtype were subdivided into different profiles by the MLST scheme, as exemplified by the 13 different profiles found among the 25 samples having the IIaA15G2R1 subtype (ESM Table S2 in Additional file 2). However, samples that had the same MLST profile were often characterized by the presence of multiple *gp60* subtypes (ESM Table S2 in Additional file 2).

### Comparison with WGS data

In a recent study based on whole genome sequence (WGS) analysis, two *C. parvum* populations were identified in Europe, one of which (population 3) has recently emerged to become predominant in Europe and the USA [17]. We therefore investigated whether the MLST scheme can distinguish between these two populations in the Italian dataset. We found that the WGS samples representing the ancestral population 2 (IT-C8, IT-C366, IT-C392, IT-C395, IT-L3, and IT-L4) and the derived population 3 (IT-C6, IT-C7, IT-C9, IT-C10, IT-C12, IT-C13, IT-C320, IT-C390, IT-C391, IT-C393, IT-G385, and IT-L1) are distinguished in the phylogenetic tree (ESM Fig. S1 in Additional file 3) and in the haplotype network (Fig. 2). Moreover, samples from population 3, including the 29 non-WGS samples typed in this work, belonged only to the *gp60* family IIa, whereas those from population 2, including the 17 non-WGS samples typed in this work, belonged to both *gp60* families IIa and IId. This result aligned with that obtained by WGS analyses and showed that, although based on a small number of SNPs, the MLST scheme can classify samples into populations 2 and 3.

## Discussion

A recently developed multi-locus sequence typing (MLST) scheme, which is based on amplification and sequencing of eight genetically unlinked markers, has been tested on a large number (> 400) of *C. parvum* samples from the four main hosts (human, cattle, sheep, and goat) and across Europe [11]. However, a clear focus on samples from different host species and from a single country was not the goal of that study, and this has limited full appreciation of the advantages and limits of the scheme in different epidemiological settings. Here, we assessed the usefulness of the new MLST scheme for molecular epidemiologic studies in Italy, a country where *C. parvum* is known to circulate in the animal reservoir and in the environment and associated with human cases [4, 5].

To this end we analyzed genotyping data from 24 human- and 48 ruminant-derived *C. parvum* samples collected across the country during 2000–2025. While the sequencing effort identified only one new allele at marker 2 in a human-derived sample, the multi-allelic profiles were largely specific of Italian samples, as 30 of the 39 profiles (76%) had not been identified in > 400 samples from other European countries so far (ESM Table S2 in Additional file 2). This result is compatible with a model of isolation by distance, as already suggested by the analysis of loci containing repetitive sequences in different European countries, including Italy [18]. Future studies in other countries are needed to confirm this model, which has important implications for molecular epidemiological investigations.

The limited information available suggested that zoonotic transmission of *C. parvum* in Italy is important and identified lambs and foals as sources of human cases, with the involvement of *gp60* subtypes IIaA20G2R1 and IIdA23G1, respectively [5, 19]. We thus examined the distribution of multi-allelic profiles of Italian samples and found that four profiles were shared by humans and animals (Fig. 1; ESM Table S2 in Additional file 2). Of note, these profiles were previously found in multiple hosts and multiple European countries (ESM Table S2 in Additional file 2) [11], therefore raising the hypothesis that some genetic variants of *C. parvum* pose a greater zoonotic risk than others.

In a previous study from Italy, 173 *C. parvum* samples collected from humans and young ruminants were genotyped using seven mini- and microsatellite loci to reveal an extensive genetic diversity, with 102 different profiles identified [4]. This study suggested that profiles from goat samples were different from those from cattle and sheep, pointing to the existence of host-adapted subpopulations. Furthermore, most samples collected before year 2000 had profiles that differed from those of samples collected after year 2000, suggesting the introduction of new genotypes over time. In the present work, we reconsidered these observations on the basis of MLST data but found no support for a goat-specific cluster (or other host-associated clusters; Fig. 1), nor for an influence of the year of collection on samples clustering (ESM Fig. S3 in Additional file 5). This is probably due to the slower rate of nucleotide substitutions in coding sequences compared with the faster accumulation of expansions/contractions of simple sequence repeats caused by slippage during DNA replication. This hypothesis can be tested by analyzing the same set of samples by the two typing schemes.

The analysis of *gp60* sequence data confirmed the high level of polymorphism that characterizes this gene [20], which identified 26 different subtypes among the Italian samples. However, while the *gp60* gene undoubtedly provided good discrimination, it is known to be a hotspot for recombination [21] and therefore cannot be used as a valid surrogate for multi-locus typing methods [22].

Comparison of samples at the whole genome level clearly represents the most accurate approach to establish their relatedness, describe population structure, and understand evolutionary aspects of *C. parvum* [16, 17] but is still difficult to apply systematically. In the largest comparative genomics study of *C. parvum* conducted in Europe, we identified two populations and showed that one has recently emerged to become dominant in both humans and animals [17]. Here, we investigated whether the MLST scheme can distinguish samples belonging to these populations and found this to be the case (Fig. 2), even using the small number of SNPs that characterized the eight gene fragments in the scheme.

This study has several limitations. First, the samples tested were not collected specifically for this study, but rather represented a convenience sampling, which limits an accurate comparison of profiles from epidemiologically linked cases versus sporadic ones. Second, the dataset has a limited size, and inferences about the zoonotic potential and population structure shall be considered as working hypotheses.

## Conclusions

This study showed that, while sequencing multiple gene markers from *C. parvum* samples collected in Italy did not reveal additional genetic variability compared with that recently described at the European scale, most of the multi-allelic profiles were restricted to Italy. This result is consistent with a model of isolation by distance that implies a significant geographical segregation of *C. parvum* in different countries.

## Supplementary Information

Additional File 1: Fig. S1. Phylogenetic analysis of *C. parvum* samples from Italy. The tree was constructed using the neighbor joining method and Tamura 3-parameter model implemented in the MEGA software version 11. Colors are used to indicate the *C. parvum* host (purple, human; red, lamb; green, goat; orange, cattle). Samples belonging to population 2, as identified by whole genome analysis, are indicated by an oval, while those belonging to population 3 are boxed.

Additional File 2: Fig. S2. Haplotype network showing the distribution of the 39 MLSTs found in 72 *C. parvum* samples from Italy. Samples are labelled by their geographical origin, with colors used to distinguish Northern Italy (yellow), Central Italy (red), and Southern Italy (pale blue).

Additional File 3: Fig. S3. Haplotype network showing the distribution of the 39 MLSTs found in 72 *C. parvum* samples from Italy. Samples are labelled by their year of collection, with colors used to indicate the different time periods considered.

Additional File 4: Table S1. List of samples included in the study, with available metadata

Additional File 5: Table S2. List of the alleles found in the 72 samples from Italy at the eight markers comprised in the MLST scheme

## Data Availability

The data supporting the findings of the study are available within the article and/or its supplementary materials. The new sequence generated in the present study was deposited in the GenBank public repository database under accession number PX516939.
