# Supplementary material for: Population structure and zoonotic potential of Cryptosporidium parvum in Italy inferred using a multi-locus sequence typing scheme
Source: Parasit Vectors. 2026 Jan 24;19:86. doi: 10.1186/s13071-025-07236-6 (PMC12911039; doi:10.1186/s13071-025-07236-6)

Figure S1. Phylogenetic analysis of *C. parvum* samples from Italy. The tree was constructed using the Neighbor Joining method and Tamura 3-parameter model implemented in the MEGA software version 11. Samples belonging to population 2, as identified by whole genome analysis, are highlighted by an oval, while those belonging to population 3 are boxed. Colors are used to indicate the *C. parvum* host (purple, human; red, lamb; green, goat, orange, cattle).


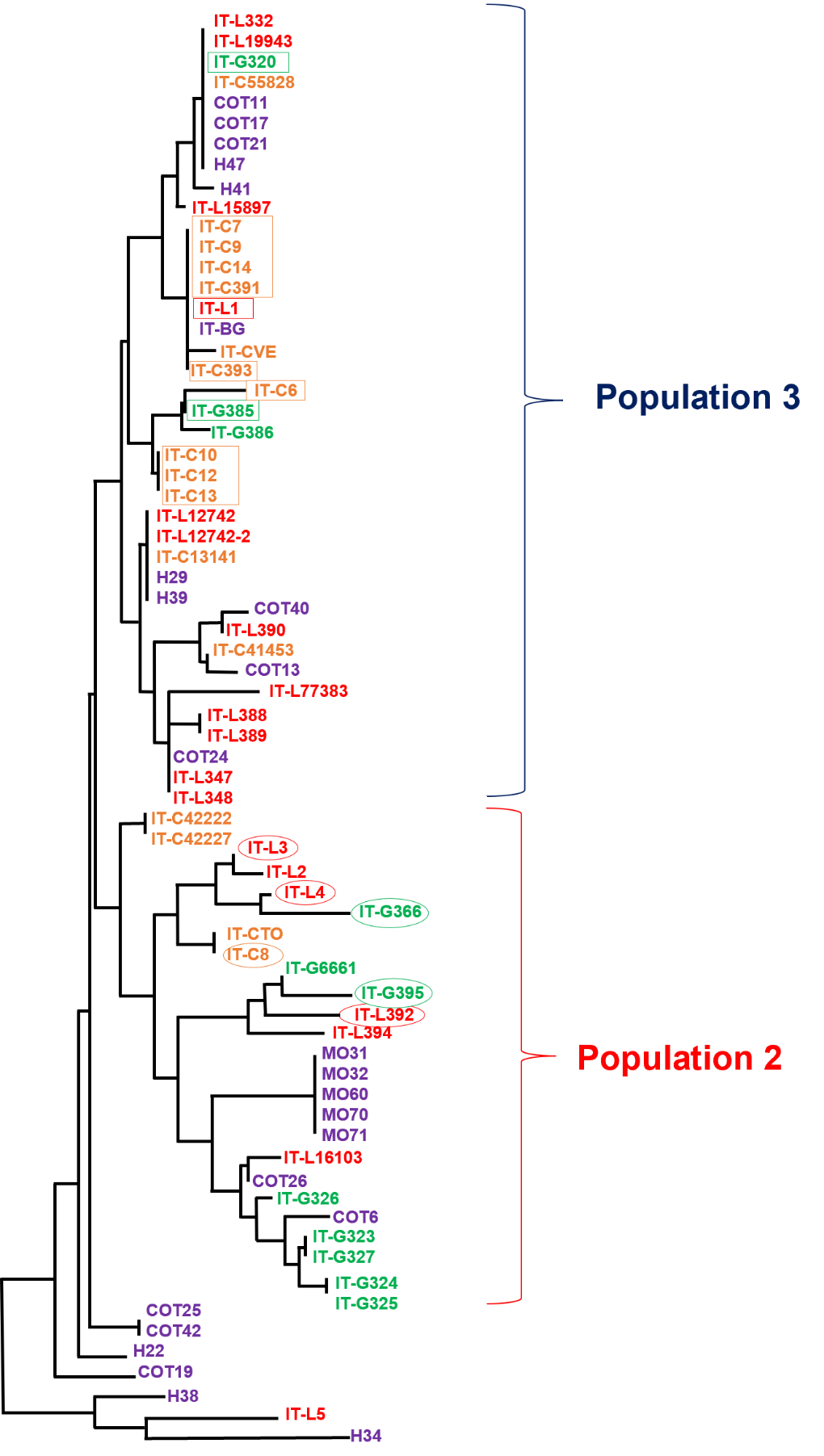

Supplement: Supplementary file 1 — Additional File 1: Fig. S1. Phylogenetic analysis of C. parvum samples from Italy. The tree was constructed using the neighbor joining method and Tamura 3-parameter model implemented in the MEGA software version 11. Colors are used to indicate the C. parvum host (purple, human; red, lamb; green, goat; orange, cattle). Samples belonging to population 2, as identified by whole genome analysis, are indicated by an oval, while those belonging to population 3 are boxed. [file 13071_2025_7236_MOESM1_ESM.docx]
