# Supplementary figures and images for: Population structure and zoonotic potential of Cryptosporidium parvum in Italy inferred using a multi-locus sequence typing scheme
Source: Parasit Vectors. 2026 Jan 24;19:86. doi: 10.1186/s13071-025-07236-6 (PMC12911039; doi:10.1186/s13071-025-07236-6)

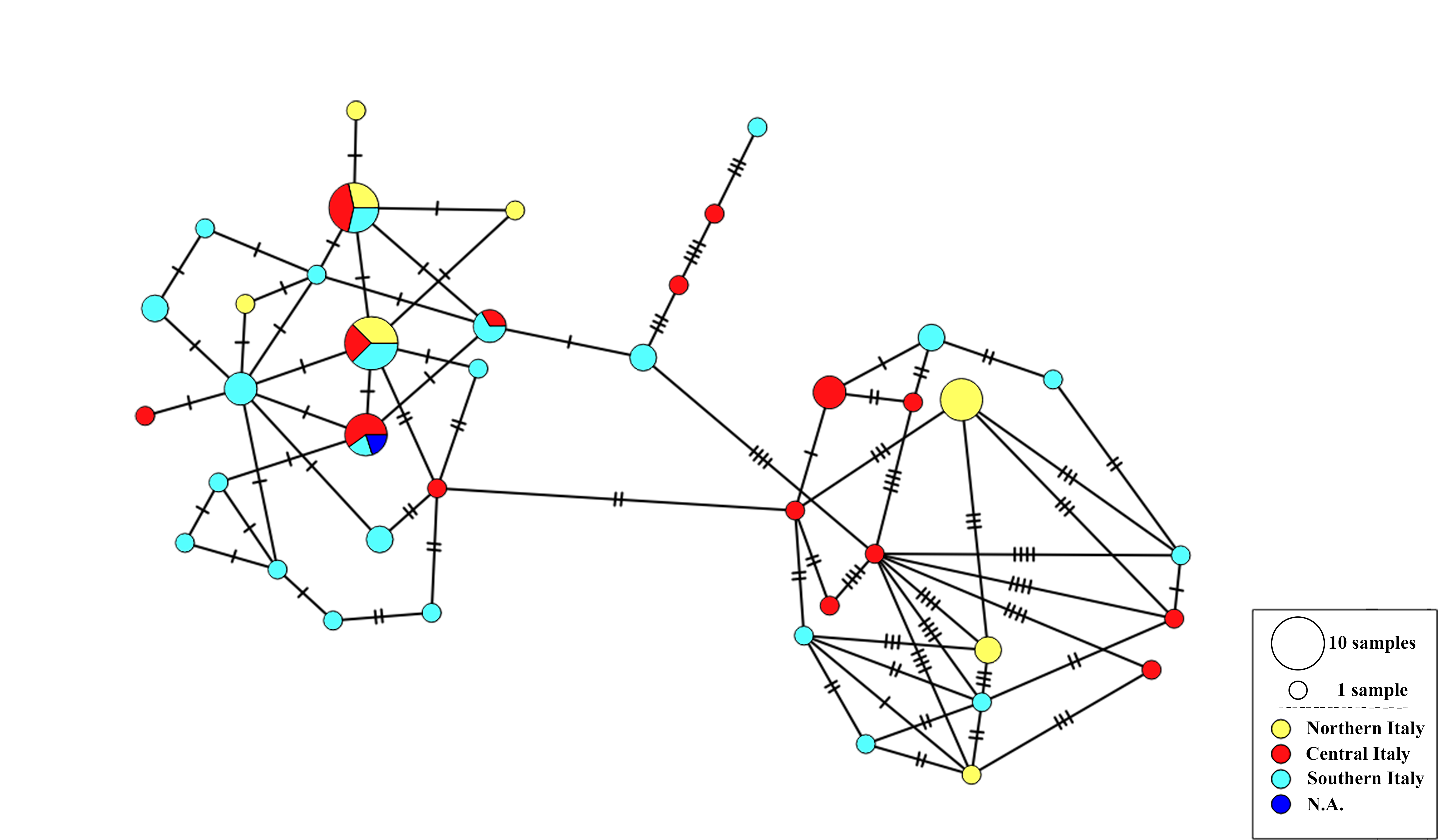

Supplement: Supplementary file 2 — Additional File 2: Fig. S2. Haplotype network showing the distribution of the 39 MLSTs found in 72 C. parvum samples from Italy. Samples are labelled by their geographical origin, with colors used to distinguish Northern Italy (yellow), Central Italy (red), and Southern Italy (pale blue). [file 13071_2025_7236_MOESM2_ESM.png]

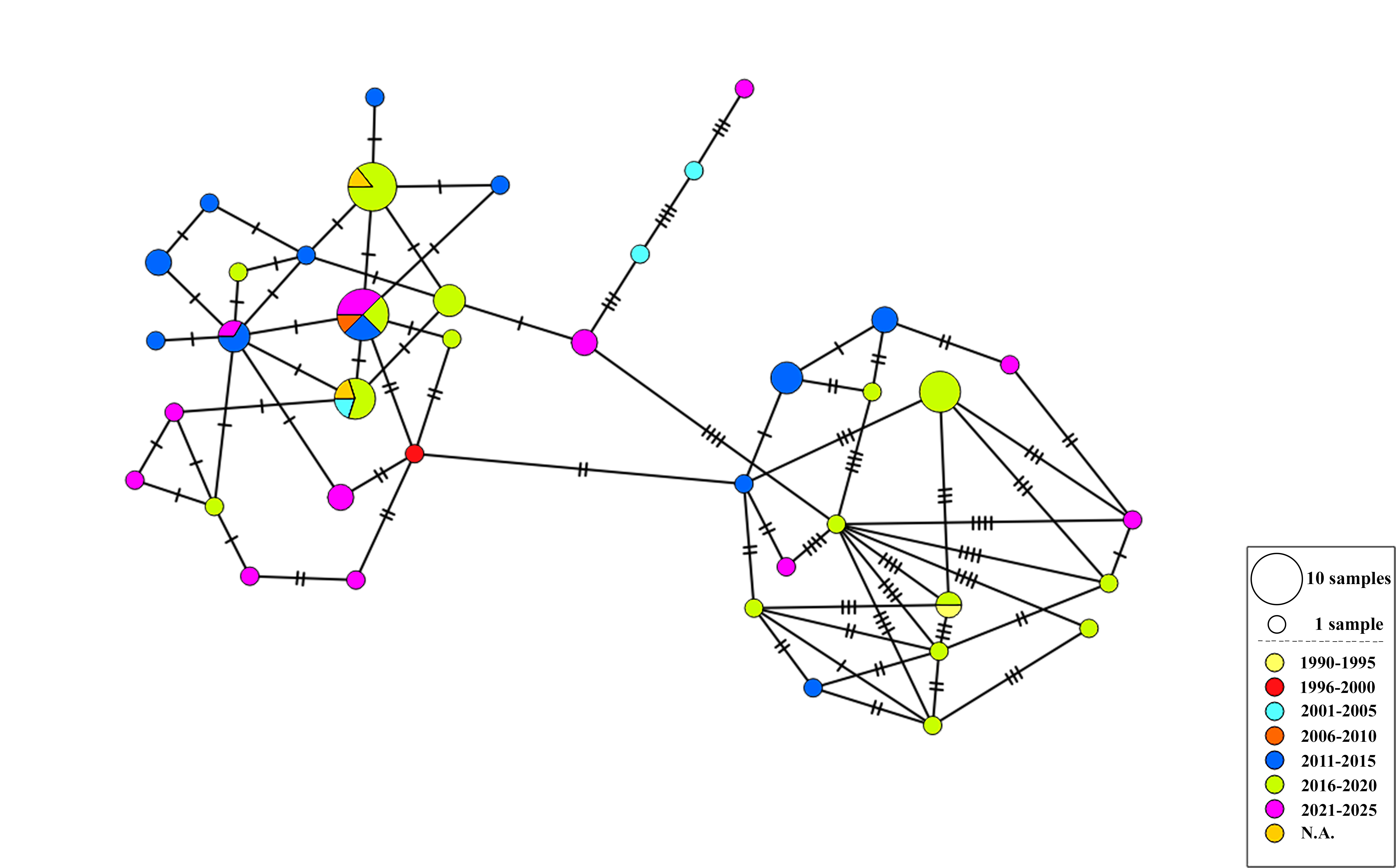

Supplement: Supplementary file 3 — Additional File 3: Fig. S3. Haplotype network showing the distribution of the 39 MLSTs found in 72 C. parvum samples from Italy. Samples are labelled by their year of collection, with colors used to indicate the different time periods considered. [file 13071_2025_7236_MOESM3_ESM.png]
