# Supplementary material for: Population structure and zoonotic potential of Cryptosporidium parvum in Italy inferred using a multi-locus sequence typing scheme
Source: Parasit Vectors. 2026 Jan 24;19:86. doi: 10.1186/s13071-025-07236-6 (PMC12911039; doi:10.1186/s13071-025-07236-6)
Supplement: Supplementary file 4 — Additional File 4: Table S1. List of samples included in the study, with available metadata [file 13071_2025_7236_MOESM4_ESM.docx]

Table S1. List of samples included in the study, with available metadata.

| Sample code | Host | Region of collection | Province of collection | Year of collection | Reference |
| --- | --- | --- | --- | --- | --- |
| BG | Human | Lombardia | Bergamo | 2014 | This study |
| COT6 | Human | Campania | Napoli | 2023 | This study |
| COT11 | Human | Campania | Napoli | 2023 | This study |
| COT13 | Human | Campania | Napoli | 2023 | This study |
| COT17 | Human | Campania | Napoli | 2023 | This study |
| COT19 | Human | Campania | Napoli | 2023 | This study |
| COT21 | Human | Campania | Napoli | 2024 | This study |
| COT22 | Human | Campania | Napoli | 2024 | This study |
| COT24 | Human | Campania | Napoli | 2024 | This study |
| COT25 | Human | Campania | Napoli | 2024 | This study |
| COT40 | Human | Campania | Napoli | 2024 | This study |
| COT42 | Human | Campania | Napoli | 2025 | This study |
| H22 | Human | Lazio | Roma | 2000 | Drumo et al (2012) |
| H29 | Human | Lazio | Roma | 2001 | Drumo et al (2012) |
| H34 | Human | Lazio | Roma | 2003 | Drumo et al (2012) |
| H35 | Human | unknown | unknown | 2003 | This study |
| H36 | Human | Lombardia | Bergamo | 2003 | This study |
| H37 | Human | Lombardia | Bergamo | 2003 | This study |
| H38 | Human | Lazio | Roma | 2004 | Drumo et al (2012) |
| H39 | Human | unknown | unknown | unknown | This study |
| H41 | Human | Lombardia | Bergamo | 2005 | Drumo et al (2012) |
| H43 | Human | unknown | unknown | 2005 | This study |
| H44 | Human | Lazio | Roma | 2005 | This study |
| H47 | Human | Lazio | Roma | 2008 | Drumo et al (2012) |
| H49 | Human | unknown | unknown | unknown | This study |
| H50 | Human | Toscana | Grosseto | 2011 | Sannella et al (2013) |
| MO32 | Human | Emilia Romagna | Modena | 2019 | Franceschelli et al (2022) |
| MO34 | Human | Emilia Romagna | Modena | 2019 | Franceschelli et al (2022) |
| MO60 | Human | Emilia Romagna | Modena | 2019 | Franceschelli et al (2022) |
| MO70 | Human | Emilia Romagna | Modena | 2019 | Franceschelli et al (2022) |
| MO71 | Human | Emilia Romagna | Modena | 2019 | Franceschelli et al (2022) |
| IT-C6 | Cattle | Piemonte | Cuneo | 2020 | Bellinzona et al (2024) |
| IT-C7 | Cattle | Lombardia | Brescia | 2020 | Bellinzona et al (2024) |
| IT-C8 | Cattle | Piemonte | Cuneo | 2020 | Bellinzona et al (2024) |
| IT-C9 | Cattle | Umbria | Perugia | 2020 | Bellinzona et al (2024) |
| IT-C10 | Cattle | Calabria | Catanzaro | 2020 | Bellinzona et al (2024) |
| IT-C12 | Cattle | Puglia | Bari | 2020 | Bellinzona et al (2024) |
| IT-C13 | Cattle | Marche | Macerata | 2020 | Bellinzona et al (2024) |
| IT-C14 | Cattle | Umbria | Perugia | 2020 | Bellinzona et al (2024) |
| IT-C13141 | Cattle | Basilicata | Matera | 2019 | This study |
| IT-C391 | Cattle | Calabria | Vibo Valentia | 2016 | Corsi et al. (2022) |
| IT-C393 | Cattle | Calabria | Vibo Valentia | 2016 | Corsi et al. (2022) |
| IT-C41453 | Cattle | Puglia | Bari | 2022 | This study |
| IT-C42222 | Cattle | Calabria | Cosenza | 2022 | This study |
| IT-C42227 | Cattle | Calabria | Cosenza | 2022 | This study |
| IT-C55828 | Cattle | Piemonte | Cuneo | 2019 | This study |
| IT-CTo | Cattle | Piemonte | Torino | 1990 | Corsi et al. (2022) |
| IT-CVe | Cattle | Veneto | Venezia | 2015 | Corsi et al. (2022) |
| IT-G320 | Goat | Emilia Romagna | Forlì | 2013 | Corsi et al. (2022) |
| IT-G323 | Goat | Marche | Macerata | 2013 | This study |
| IT-G324 | Goat | Calabria | Cosenza | 2013 | This study |
| IT-G325 | Goat | Calabria | Cosenza | 2013 | This study |
| IT-G326 | Goat | Marche | Macerata | 2013 | This study |
| IT-G327 | Goat | Marche | Macerata | 2013 | This study |
| IT-G366 | Goat | Basilicata | Matera | 2014 | Corsi et al. (2022) |
| IT-G385 | Goat | Campania | Salerno | 2015 | Corsi et al. (2022) |
| IT-G386 | Goat | Campania | Salerno | 2015 | Corsi et al. (2022) |
| IT-G395 | Goat | Umbria | Terni | 2016 | Corsi et al. (2022) |
| IT-G6661 | Goat | Marche | Macerata | 2022 | This study |
| IT-L1 | Sheep | Toscana | Grosseto | 2020 | Bellinzona et al (2024) |
| IT-L2 | Sheep | Piemonte | Torino | 2020 | Bellinzona et al (2024) |
| IT-L3 | Sheep | Basilicata | Potenza | 2020 | Bellinzona et al (2024) |
| IT-L4 | Sheep | Basilicata | Potenza | 2020 | Bellinzona et al (2024) |
| IT-L5 | Sheep | Calabria | Cosenza | 2020 | Bellinzona et al (2024) |
| IT-L12742 | Sheep | Umbria | Perugia | 2019 | This study |
| IT-L12742-2 | Sheep | Umbria | Perugia | 2019 | This study |
| IT-L15897 | Sheep | Sardegna | Sassari | 2019 | This study |
| IT-L16103 | Sheep | Marche | Ancona | 2019 | This study |
| IT-L19943 | Sheep | Piemonte | Cuneo | 2019 | This study |
| IT-L332 | Sheep | Umbria | Perugia | 2012 | This study |
| IT-L347 | Sheep | Basilicata | Potenza | 2013 | This study |
| IT-L348 | Sheep | Basilicata | Potenza | 2013 | This study |
| IT-L388 | Sheep | Campania | Salerno | 2015 | Corsi et al. (2022) |
| IT-L389 | Sheep | Campania | Salerno | 2015 | Corsi et al. (2022) |
| IT-L390 | Sheep | Campania | Salerno | 2016 | Corsi et al. (2022) |
| IT-L392 | Sheep | Toscana | Grosseto | 2016 | Corsi et al. (2022) |
| IT-L394 | Sheep | Umbria | Terni | 2016 | Corsi et al. (2022) |
| IT-L77383* | Sheep | Toscana | Grosseto | 2011 | Sannella et al. (2013) |

*, this sample consisted of a microscopy slide prepared for the analysis of a sheep fecal sample (Sannella et al., 2013).
